# Characterization of Human Coronavirus Etiology in Chinese Adults with Acute Upper Respiratory Tract Infection by Real-Time RT-PCR Assays

**DOI:** 10.1371/journal.pone.0038638

**Published:** 2012-06-15

**Authors:** Roujian Lu, Xiaoyan Yu, Wen Wang, Xijie Duan, Linglin Zhang, Weimin Zhou, Jin Xu, Lingjie Xu, Qin Hu, Jianxin Lu, Li Ruan, Zhong Wang, Wenjie Tan

**Affiliations:** 1 National Institute for Viral Disease Control and Prevention, China CDC, Beijing, China; 2 Peking Union Medical College Hospital, Chinese Academy Medical Sciences, Beijing, China; 3 Key Laboratory of Laboratory Medicine, Ministry of Education, and Institute of Medical Virology, Wenzhou Medical College, Zhejiang, China; Faculty of Biochemistry Biophysics and Biotechnology, Jagiellonian University, Poland

## Abstract

**Background:**

In addition to SARS associated coronaviruses, 4 non-SARS related human coronaviruses (HCoVs) are recognized as common respiratory pathogens. The etiology and clinical impact of HCoVs in Chinese adults with acute upper respiratory tract infection (URTI) needs to be characterized systematically by molecular detection with excellent sensitivity.

**Methodology/Principal Findings:**

In this study, we detected 4 non-SARS related HCoV species by real-time RT-PCR in 981 nasopharyngeal swabs collected from March 2009 to February 2011. All specimens were also tested for the presence of other common respiratory viruses and newly identified viruses, human metapneumovirus (hMPV) and human bocavirus (HBoV). 157 of the 981 (16.0%) nasopharyngeal swabs were positive for HCoVs. The species detected were 229E (96 cases, 9.8%), OC43 (42 cases, 4.3%), HKU1 (16 cases, 1.6%) and NL63 (11 cases, 1.1%). HCoV-229E was circulated in 21 of the 24 months of surveillance. The detection rates for both OC43 and NL63 were showed significantly year-to-year variation between 2009/10 and 2010/11, respectively (P<0.001 and P = 0.003), and there was a higher detection frequency of HKU1 in patients aged over 60 years (P = 0.03). 48 of 157(30.57%) HCoV positive patients were co-infected. Undifferentiated human rhinoviruses and influenza (Flu) A were the most common viruses detected (more than 35%) in HCoV co-infections. Respiratory syncytial virus (RSV), human parainfluenza virus (PIV) and HBoV were detected in very low rate (less than 1%) among adult patients with URTI.

**Conclusions/Significance:**

All 4 non-SARS-associated HCoVs were more frequently detected by real-time RT-PCR assay in adults with URTI in Beijing and HCoV-229E led to the most prevalent infection. Our study also suggested that all non-SARS-associated HCoVs contribute significantly to URTI in adult patients in China.

## Introduction

Human coronaviruses (HCoVs) are enveloped viruses with a single-strand RNA genome [1]. 5 species are known to infect humans, 229E and OC43 first were identified in the 1960s, NL63 and HKU1 identified in 2004 and 2005, respectively [1]–[3], and SARS-CoV was identified during the severe acute respiratory syndrome epidemic in 2003 [4]–[5]. HCoVs are associated with respiratory syndromes ranging from mild upper to severe lower respiratory tract infections including pneumonia and bronchiolitis [1], [6]–[9]. Specifically, HCoVs can elicit a more serious respiratory disease in children, the elderly and people with underlying disease [1], [6], [10]–[11]. HCoVs circulate worldwide, although their frequencies differ from country to country depending on seasonality and detection annum [1], [12]–[16]. Most studies have focused on children or adults with more serious respiratory disease including lower respiratory tract infections [1], [17]–[22].

Upper respiratory tract infections (URTI) or “the common cold” are a significant health burden, especially among children. The major viral agents of URTI in children include rhinoviruses, HCoVs (–OC43 and –229E), RSV,PIV, adenovirus(ADV) and influenza(Flu). newly identified viruses (hMPV, HCoV-NL63,HCoV-HKU1 and HBoV) are also included. Although HCoVs are recognized as one of the most frequent causes of URTI or common colds in adults [7], epidemiological data and clinical profiles are limited on HCoVs infection in adults with URTI continuously for several years with sensitive molecular methods [1], [13], [20], [23]–[25], especially for HCoV-NL63 and HCoV-HKU1 in China after 2009 H1N1 pandemic. Ren et al reported the prevalence of HCoVs was 1% in adults with ARTI in Beijing from 2005 to 2009 by RT-PCR assays [24], which was significantly lower than in previous literatures indicated above 2.1% [1], [13], [20], [23]. In the present study, 981 nasopharyngeal swabs were collected continuously from adults with URTI in Beijing between March 2009 and February 2011. HCoVs infections were detected by Real-Time RT-PCR with excellent specifity and sensitivity [19], [23], [26], other 11 respiratory viruses infection among all specimens were also detected by sensitive molecular assays [25]–[28]. HCoVs infection and their clinical and epidemiological characteristics were analyzed.

## Materials and Methods

### Ethics Issues

All aspects of the study were performed in accordance with the national ethics regulations and approved by the Institutional Review Boards of the Centre for Disease Control and Prevention of China, as well as the Ethics Committee of Peking Union Medical College Hospital. Participants were received "Written Informed Consent" on the study’s purpose and of their right to keep information confidential. Written consent was obtained from all participants or their guardians.

### Patients and Specimens

Nasopharyngeal swabs were collected from adults presenting with URTI to the Peking Union Medical College Hospital, Beijing, China, between March 2009 and February 2011. Patients provided informed consent for specimen collection and testing. All patients over 14 years of age were selected according to a set of criteria that included respiratory symptoms, a body temperature above 37.5°C, and a normal or low leukocyte count(≤10^10^/L), but not LRTI identified by pulmonary abnormalities on radiography and clinical descriptions(without bronchitis, bronchiolitis and pneumonia). Demographic data and clinical findings at the time of diagnosis were recorded on a standard form. All swabs were collected into viral transport medium and transported for immediate testing to the National Institute for Viral Disease Control and Prevention (NIVDC), China Center for Disease Control and Prevention.

### Nucleic Acid Extraction and cDNA Synthesis

Viral RNA was extracted from 200 µl of sample using QIAamp MinElute Virus Spin kits (Qiagen, Germany). cDNA was synthesized with AMV reverse transcriptase and random hexamer primers (Promega, USA) as described previously [19], [23], [25].

### Real-time RT-PCR for HCoV Detection

Real-time RT-PCR amplification was performed using TaqMan® One-Step RT-PCR Master Mix Reagents Kit (Applied Biosystems, USA) [19], [23], [26]. A real-time reverse transcription (RT)-PCR technique was used to screen for each of the 4 non-SARS HCoVs (Table 1). PCR was performed under the following conditions: 48°C for 30 min, 95°C for 15 min, followed by 40 cycles at 95°C for 15s, 68°C for 1min.The lower limit of detection of each HCoV real-time RT-PCR assay was 50–100 copies/25uL.The assay sensitivity, specificity and coefficients of variability(CVs) were evaluated and validated as previously described [19], [26].

**Table 1:** Primers and Probes (5′–3′), and Their Gene Product Targets, Used for the Respiratory Viruses Identified in the Study.

Assays andViruses detected		Primer* and probe	Target genes	Ref
**Real-time rtPCR**				
HCoV-OC43	OC43-F	GCTCAGGAAGGTCTGCTCC	N	19
	OC43-R	TCCTGCACTAGAGGCTCTGC		
	OC43-P	FAM –TTCCAGATCTACTTCGCGCACATCC-TAMRA		
HCoV-229E	229E-F	CGCAAGAATTCAGAACCAGAG	N	19
	229E-R	GGCAGTCAGGTTCTTCAACAA		
	229E-P	FAM –CCACACTTCAATCAAAAGCTCCCAAATG-TAMRA		
HCoV-NL63	NL63-F	AGGACCTTAAATTCAGACAACGTTCT	N	19
	NL63-R	GATTACGTTTGCGATTACCAAGACT		
	NL63-P	FAM- TAACAGTTTTAGCACCTTCCTTAGCAACCCAAACA-TAMRA		
HCoV-HKU1	HKU1-F	AGTTCCCATTGCTTTCGGAGTA	N	19
	HKU1-R	CCGGCTGTGTCTATACCAATATCC		
	HKU1-P	FAM -CCCCTTCTGAAGCAA- MGB		
**Multiple-nested PCR**				
**Mix1** FluA	FA-1F	CAGAGACTTGARRATGTYTTTGC	Matrix	26
	FA-1R	GGCAAGYGCACCRGYWGARTARCT		
	FA-2F	GACCRATCCTGTCACCTCTGACT		
	FA-2R	AYYTCYTT GC CCATGGAATGT		
FluB	FB-1F	GTGACTGGTGTGATACCACT	HA	
	FB-1R	TGTTTTCACCCATATTGGGC		
	FB-2F	CATTTTGCAAATCTCAAAGG		
	FB-2R	TGGAGGCAATCTGCTTCACC		
ADV	AD-1F	GCCGCAGTGGTCTTACATGCACATC	Hexon	
	AD-1R	CAGCACGCCGCGGATGTCAAAGT		
	AD-2F	GCCACCGAGACGTACTTCAGCCTG		
	AD-2R	TTGTACGAGTACGCGGTATCCTCGCGGTC		
	AD-2F9	CMGASACSTACTTCAGYMTG		
	AD-2R9	GTASGYRKTRTCYTCSCGGTC		
**Mix2** hRSV	RS-1F	TGGGAGARGTRGCTCCAGAATACAGGC	N	26
	RS-1R	ARCATYACTTGCCCTGMACCATAGGC		
	RS-2F	ACYAAATTAGCAGCAGGG		
	RS-2R	CTCTKGTWGAWGATTGTGC		
Picornavirus	PIC-1F	GCACTTCTGTTTCCCC	5?-UTR	
	PIC-1R	CGGACACCCAAAGTAG		
	PIC-2F	GCACTTCTGTTTCCCC		
**Mix3** PIV (-1,-2,-3)	P123-1F	GTWCAAGGAGAYAATCARGC	L	26
	P123-1R	GRTCYGGAGTTTCWARWCC		
	P1-2F	GCATCAGACCCTTATTCATG		
	P1-2R	GTTGTATCAAGCATCCCGGC		
	P2-2F	CAGCCGATCCATACTCATTG		
	P2-2R	CTTGTGGTGTCAAAAAATCC		
	P3-2F	GCTGTTACTACAAGAGTACC		
	P3-2R	GTTGCCAGATTTGAGGATGC		
**rtPCR**				
hMPV	hMPV-F	AACCGTGTACTAAGTGATGCACTC	N	27
	hMPV-R	CATTGTTTGACCGGCCCCATAA		
**Nested PCR**				
HBoV	HBoV-1F	CCAGCAAGTCCTCCAAACTCACCTGC	NP-1	28
	HBoV-1R	GGAGCTTCAGGATTGGAAGCTCTGTG		
	HBoV-2F	GACCTCTGTAAGTACTATTAC		
	HBoV-2R	CTCTGTGTTGACTGAATACAG		

* K  =  G+T, M  =  A+C, R  =  A+G, S  =  G+C, W  =  A+T, Y  =  C+T.

1st round primers:–1F,–1R; 2 nd round primers: –2F or –2F9, –2R or –2R9.

### Gel-based RT-PCR for Detection of non-HCoV Respiratory Viruses

Specimens were also screened for influenza virus types A and B, parainfluenza virus types 1 to 3, respiratory syncytial virus (RSV), picornaviruses (including enteroviruses and rhinoviruses) and adenoviruses using three multiple-nested PCR assays [25]–[26] (Table 1), for human metapneumovirus (hMPV) and human bocavirus (HBoV) using PCR and nested-PCR assays [26]–[28] (Table 1). Multiple-nested PCR and nested PCR programs were run as follows: 1^st^ round: 94°C for 2 min; 94°C for 30 s, 55/50°C 30 s, 72°C for 1 min, 35 cycles; 72°C 5 min. 2^nd^ round: 94°C for 2 min; 94°C for 30s, 55/50°C 30s, 72°C for 1 min, 25 cycles; 72°C 5 min. RT-PCR program was run as follows: 50°C 30 min; 95 °C 15 min; 95°C 30s, 60°C 30s, 72°C 1 min, 40 cycles; 72°C 5 min. The lower limits of detection for nested PCR and RT-PCR assays were 10–100 single virus molecules/25 ul. All PCR products were confirmed by sequencing.

### Statistical Analysis

Statistical analysis was performed using the predictive analysis software (PASW) statistics 18 package with X^2^-test and Fisher’s exact test.

## Results

### Detection of HCoVs and Other 11 Respiratory Viruses Infection

A total of 981 nasopharyngeal swabs were collected. The male: female ratio was 438∶543 (1∶1.24) and the median age was 29 years (age range 14 years to 91 years). HCoVs were detected by real-time RT-PCR in 157 (16.0%) of the 981 specimens: 229E in 96 (9.8%), OC43 in 42 (4.3%), HKU1 in 16 (1.6%) and NL63 in 11(1.1%). The detection rates of HCoVs are summarized in Table 2. Differences in the detection rates of OC43 and NL63 during 2009/10 and 2010/11 were statistically significant (P<0.001 and P = 0.003, respectively).

**Table 2:** Prevalence of Different Respiratory Virus Infections from March 2009 to February 2011.

Respiratory Virus		No. of detections (%)		Total
		03/09–02/10	03/10–02/11	
		(N = 539 cases)	(N = 442 cases)	981
HCoV-OC43		38(7.1)**	4(0.9)	42(4.3)
HCoV-229E		58(10.8)	38(8.6)	96(9.8)
HCoV-NL63		11(2.0) *	0(0)	11(1.1)
HCoV-HKU1		12(2.2)	4(0.9)	16(1.6)
Flu A		84(15.6) **	3(0.7)	87(8.9)
Flu B		17(3.2)	1(0.2)	18(1.8)
PIV-1		4(0.7)	1(0.2)	5(0.5)
PIV-2		1(0.2)	0(0)	1(0.1)
PIV-3		0(0)	0(0)	0(0)
ADV		19(3.5)	1(0.2)	20(2.0)
hRSV		1(0.2)	0(0)	1(0.1)
Piconavirus	Rhinoviruses	57(10.6)	14(3.2)	71(7.2)
	Enterovirus	7(1.3)	0(0)	7(0.7)
hMPV		13(2.4)	13(2.9)	26(2.7)
HBoV		0(0)	2(0.5)	2(0.2)

** P<0.001; *P = 0.003.

Other 11 respiratory viruses infection among all specimens were also detected by sensitive molecular assays (Table 1). During the same period, Flu A virus was detected in 87(8.9%) patients, Flu B virus was detected in 18(1.8%) patients, PIV-1 was detected in 5(0.5%) patients, PIV-2 was detected in 1(0.1%) patient, ADV was detected in 20(2.0%) patients, RSV was 1(0.1%) patient, rhinoviruses was detected in 71(7.2%) patients, enterovirus was detected in 7(0.7%) patients, hMPV was detected in 26(2.7%), and HBoV was detected in 2(0.2%) patients(Table 2).

### Epidemiology of HcoVs

**Figure 1:**
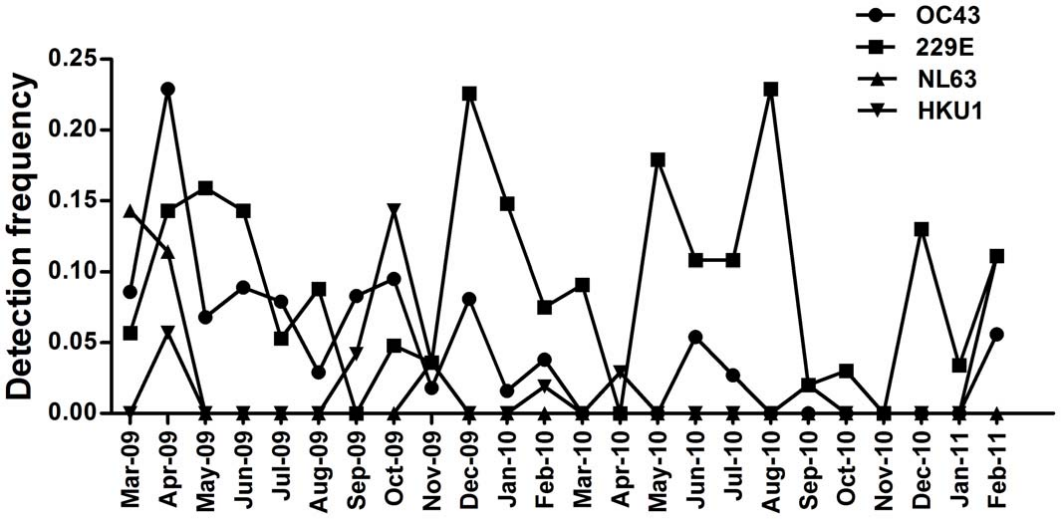
Temporal distribution of the 4 HCoV species between March 2009 and February 2011 inclusive.

The seasonal distribution of HCoVs is shown in Fig.1. HCoV-229E was the most common species, appearing in most months and without an obvious seasonal distribution. Peaks in 229E detections occurred in May 2009, December 2009 and August 2010, corresponding with spring, winter and summer, respectively, in Beijing. HCoV-OC43 detections peaked in April 2009. This virus continued to circulate for the next 10 months but only sporadic infections were detected in the second half of the study. In 2009, when most coronavirus infections occurred, 229E and OC43 were detected in every evaluable month, with the exception of 229E in September. A peak of NL63 activity occurred in March and April 2009 with little or no subsequent activity. HCoV-HKU1 activity was sporadic throughout, with no obvious seasonal distribution.

**Figure 2:**
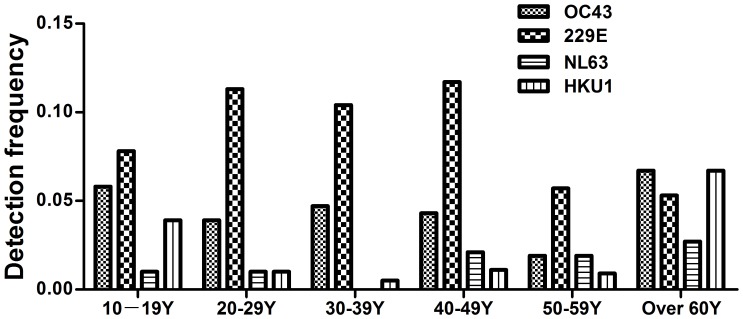
Frequencies of the four non SARS HCoVs infections detection by different age groups.

HCoVs were detected across all age groups, although NL63 cases were absent in the 30–39 years age group (Fig.2). A significantly higher detection frequency of HKU1 occurred in patients aged over 60 years (P = 0.03), but there were no significant differences in age group distribution for 229E, OC43 and NL63.

### Clinical Characteristics of Patients with HCoVs Infection

Approximately equal numbers of males and females were infected with OC43, HKU1 and NL63; 229E infected significantly more female than males (62.5% versus 37.5%, P = 0.009). The clinical characteristics are summarized in Table 3. All HCoV positive patients presented with URTI. Fever, sore throat and headache were the most common symptoms at presentation. Gastrointestinal tract associated symptoms were rare. Overall there were no statistically significant differences in the clinical symptoms between species-specific HCoVs except for rhinorrhea, which was observed less in 229E-infected patients than in the other cases (P = 0.025).

**Table 3:** Clinical characteristics of patients with HCoV infections in the study.

Characteristics	OC43 (N = 42)	229E (N = 96)	NL63 (N = 11)	HKU1(N = 16)	P-value
**Demographics:**					
Gender ratio (M/F)	1.1	0.58	0.83	0.78	
Age in years mean/median	33.5/28.5	32.3/28	41.45/41	38.1/31.5	
**Respiratory symptoms:**					
Fever(>38°C)	37 (88.1%)	79 (82.3%)	10 (90.9%)	12 (85.7%)	ns
Chills	6 (14.3%)	24 (25%)	2 (18.2%)	4 (25%)	ns
Sore throat	27 (64.3%)	63 (65.6%)	8 (72.7%)	10 (62.5%)	ns
Nasal obstruction	9 (21.4%)	18 (18.8%)	3 (27.3%)	2 (12.5%)	ns
Rhinorrhea	17 (40.5%)	23 (24%)	5 (45.5%)	9 (56.2%)	= 0.025
Cough	14 (33.3%)	30 (31.2%)	7 (63.6%)	9 (56.3%)	ns
Sputum production	8 (19%)	16 (16.7%)	5 (45.5%)	4 (25%)	ns
**Gastrointestinal symptoms:**					
Vomiting	4 (9.5%)	5 (5.2%)	1 (9.1%)	0	ns
Diarrhea	1 (2.4%)	6 (6.2%)	1 (9.1%)	0	ns
**Other symptoms:**					
Headache	30 (71.4%)	68 (70.8%)	5 (45.5%)	11 (68.8%)	ns
Muscle pain	1 (2.4%)	4 (4.2%)	0	1 (6.3%)	ns

### Co-detections of other Respiratory Virus in HCoV-positive Cases

High rates of co-infection with non-HCoV respiratory viruses were observed (Table 4). Of the 157 HCoV positive patients, 48 (30.57%) were co-infected, 38 of them with one other virus and 10 with 2 other viruses. Most HCoV-associated co-infections involved human rhinoviruses (all picornaviruses positive samples were confirmed to be rhinoviruses by DNA sequencing) and Flu A virus (more than 30%). Compared to single HCoV infections, there was no evidence to indicate that patients with co-infections had more serious illnesses (results not shown).

**Table 4:** Co-Detections of HCoVs and other Respiratory Virus in the study population.

Co-Detection withHCoVs(n = 157)	Number of cases (n = 48)
**HCoVs+ Flu A**	18
229E+ Flu A	10
NL63+ Flu A	1
HKU1+ Flu A	2
OC43+229E+ Flu A	2
OC43+HKU1+ Flu A	2
NL63+HKU1 + Flu A	1
**HCoVs +Flu B**	1
229E+Flu B	1
**HCoV+ ADV**	6
OC43+ADV	3
229E+ADV	2
NL63+ADV+rhinoviruses*	1
**HCoVs+ hRSV**	1
OC43+HKU1+hRSV	1
**HCoVs+ rhinoviruses**	18
OC43+rhinoviruses	5
229E+rhinoviruses	9
HKU1+rhinoviruses	1
OC43+NL63+rhinoviruses	1
NL63+HKU1+rhinoviruses	1
NL63+ADV+rhinoviruses*	1
**HCoVs+hMPV**	1
229E+hMPV	1
**HCoVs+HCoVs**	4
OC43+229E	3
OC43+229E+NL63	1

* Be only counted once in the total.

## Discussion

HCoVs circulate worldwide and the prevalence of the different species varies by year and geographical location [1]. Recent reports show that HCoVs have been detected in Hong Kong [29], Italy [13], France [22], [30], USA [18], [21], [31], UK [23], Netherlands [19], Finland [32], and China [24]–[25] in respiratory and/or stool samples obtained from young children [18], [21]–[22], [31] and adults [20], [23]–[24], [33]. In our retrospective study, a 16.0% detection rate for HCoVs infection was found in adults with URTI; 229E was the most common infection (9.8% of all cases), followed by OC43 (4.3%), HKU1 (1.6%) and NL63 (1.1%). The detection rates for 229E and OC43 are consistent with other studies performed worldwide (0.1–26% &0.5–10.1%) [1], [11], [22], [26], [29]–[37], but higher than in a recent study undertaken in Beijing [24], perhaps due in our study to the exclusion of patients with lower respiratory tract infections via clinical descriptions and radiography, the use of a screening criteria leukocyte count of ≤10^10^/L which is likely to have excluded most patients with bacterial infection, and the use of real-time PCR with excellent sensitivity [17], [19], [23]. The studies were also undertaken in different years, suggesting that the presence or absence of circulating HCoV species during these times may have influenced these differed outcomes [1], [17], [24]. In addition, Flu A detection rate was 15.6% among URTI adult patients between 2009/2–2010/3 with the occurrence of pandemic H1N1 virus 2009, which was signicantly higher than that (0.7%) of patients between 2010/2–2011/3 (Table 2). Whether the circulating patterns of HCoVs might be affected by FluA pandemic, warrants further studies.

HCoVs exhibit variable circulation approximately every 2–3 years [1], [17], [24], [34]–[35], and this was confirmed by our observation that detection rates for both OC43 and NL63 were statistically significant between 2009/10 and 2010/11. Nevertheless, 229E strains circulated throughout the 2 years of study, often in high numbers and without discernible seasonality, in contrast to that reported in the UK [23].

A previous study in Beijing reported higher detection rates for HCoVs in individuals over 65 years of age [24]. In contrast, in this study HCoVs were detected essentially in all adult age groups. Of note, however, HKU1 infection predominated in patients aged over 60 years, suggesting that adults in this category may be at higher risk for infection with this species.

URTI occurs commonly in both children and adults and is a major cause of mild morbidity which have a high cost to society. URTI among adults are responsible for missed work and unnecessary medical care (antibiotic prescriptions), also occasionally associated serious sequelae. The clinical diagnosis of all HCoVs positive patients we studied was URTI. The main clinical signs at presentation were fever, cough, sore throat and headache, in accord with several previous studies [1], [7], [22]. Some cases also showed gastrointestinal symptoms except in HCoV-HKU1 infection patients [1], [32]. Rhinorrhea was observed in significantly fewer patients infected with 229E. Many previous reports have indicated that HCoVs have high rates of co-infection with other respiratory viruses [1], [19], [23], [26], including rhinoviruses, influenza, HMPV, ADV and RSV. In this study, co-infections were associated with each of the non-SARS HCoVs at rates similar to previous studies [1], [18], [23], [26]. Human rhinoviruses and influenza A viruses were the most common respiratory viruses detected in HCoVs-positive patients, possibly reflect the level of their circulation at the time, and which were consistent with rhinoviruses being the most common respiratory viruses for common cold [1], [36]. In contrast with virus infection among children with URTI [1], [7], [23], [36], RSV, PIV and HBoV were detected in very low rate(less than 1%) among adults with URTI in this study. It was consistent with the previous reports [26], [37]. Comparison of clinical symptoms experienced by patients with and without co-infections indicated that co-infection with HCoVs did not affect illness severity.

In summary, we found that the four non-SARS HCoVs are regularly associated with URTI in adult patients in China, although distinct seasonal patterns of circulation are not obvious. Our observation that elderly adults may be are at high risk of HKU1 infection requires further study. Our results suggest that testing algorithms that include sensitive HCoVs detection contribute to the likelihood of obtaining a laboratory diagnosis when respiratory virus infection is suspected [26], [37]–[38]. To the best of our knowledge, this is the first comprehensive analysis of the potential impact of four non-SARS HCoVs (HCoV-OC43, HCoV-229E, HCoV-NL63 and HCoV-HKU1) as causes of URTIs in adults admitted to hospital in China by real time RT-PCR assays. To identify the role of HCoVs infection in URTI of adults, more specimens include control subjects with clear clinical and epidemiological profiles warrant be investigated.
